# Factors predicting the mathematics anxiety of adolescents: a structural equation modeling approach

**DOI:** 10.3389/fpsyt.2024.1484381

**Published:** 2024-12-05

**Authors:** Suman Ahmmed, Jashodhan Saha, Maruf Ahmed Tamal, Khondaker Abdullah Al Mamun, Sajani Islam

**Affiliations:** ^1^ Department of Computer Science & Engineering, United International University, Dhaka, Bangladesh; ^2^ Institute of Natural Sciences, United International University, Dhaka, Bangladesh; ^3^ Institute for Advanced Research, United International University, Dhaka, Bangladesh; ^4^ Department of Pediatrics, Shaheed Suhrawardy Medical College, Dhaka, Bangladesh

**Keywords:** mathematics anxiety, mathematics performance, factors of mathematics anxiety, partial least squares structural equation modeling (PLS-SEM), adolescents in Bangladesh

## Abstract

**Introduction:**

Mathematics anxiety (MA) is a distinct negative emotional state or trait that individuals experience when confronted with mathematical problems in everyday life and academic contexts. This study aims to identify the key predictors of MA among secondary-level students in Bangladesh.

**Methods:**

Utilizing a quantitative cross-sectional research design, data were collected from 486 students across 89 institutions. Later, the data were analyzed using Partial Least Squares Structural Equation Modeling (PLS-SEM).

**Results:**

The findings revealed that math related negative past experiences (β = 0.241, t = 4.914, p < 0.001) and a perceived lack of teacher support (β = 0.234, t = 5.440, p < 0.001) significantly contribute to students’ low self-efficacy in mathematics. This low self-efficacy is further influenced by negative attitudes and test anxiety, ultimately leading to increased MA (β = 0.694, t = 22.695, p < 0.001). Additionally, cognitive challenges, particularly working memory difficulties, directly affect MA (β = 0.110, t = 2.659, p = 0.008). The study also found that negative attitudes (β = 0.347, t = 9.063, p < 0.001) and test anxiety (β = 0.251, t = 5.913, p < 0.001) independently exacerbate MA. Moreover, a lack of motivation in learning mathematics is directly influenced by this elevated level of MA (β = 0.384, t = 9.939, p < 0.001).

**Discussion:**

Taken together, the study proposes several key recommendations and policy implications to inform the development of synchronized policies by educational authorities aimed at combatting, reducing MA among secondary-level students in Bangladesh and similar contexts.

## 1 Introduction

Globally, anxiety disorders have emerged as the most prevalent and widespread mental health issue. According to the World Health Organization (WHO), approximately 4% of the global population currently experiences at least one anxiety disorder (1). While occasional anxiety is a normal aspect of life, it becomes problematic when it transforms into frequent, persistent worry or fear. There are various forms of anxiety disorders, including generalized anxiety disorder, social anxiety disorder, panic disorder, and phobia-related disorders etc. In educational settings, test anxiety and performance anxiety are particularly common among students. However, a distinct form of anxiety disorder, known as MA, has recently gained increasing focus as a frequent and alarming issue on a global scale in all age groups of students (2, 3). MA has been defined in various ways by researchers, educational psychologists (EPs) and practitioners from different point of view. Consequently, there is no universally accepted or rigid definition for the term MA. However, in most cases, MA is defined as a distinct negative emotional state or trait that individuals experience when confronted with mathematical problems in everyday life and academic contexts (4, 5). Beyond its negative effects on mental health and well-being, MA is well-documented for its negative correlation with mathematical competence, performance and achievement (6, 7), making it a potential barrier to success in science, technology, engineering, and mathematics (STEM) careers (8).

Though MA has short, medium, and long-term negative consequences on students’ self-confidence, math performance, math achievement, and even career choices, the specific factors and other events that trigger to the development of MA remain still unclear (9). However, existing studies broadly categorize these factors into personal, psychological, environmental, cognitive, and pedagogical influences. Among personal factors, gender has received significant attention in relation to MA. Many studies have found that females are more prone to MA than males (10–13), which would suggest that females’ math performance should also be more negatively affected. However, despite these findings, research often reveals little to no gender differences in actual math performance (14–16). Another potential factor that has been investigated under the personal factors is genetics. For instance, Malanchini et al. (17) found that 75% of the genetic variance in MA is tied to math-related attitudes, abilities, and achievement. Wang et al. (18) similarly reported that 40% of the variance in MA is genetically influenced, either directly or through contributions to mathematical ability and general anxiety. In terms of psychological factors, self-efficacy is frequently studied in connection with MA (19). For example, Khasawneh et al. (10) found a significant negative correlation between MA and self-efficacy levels, suggesting that improving students’ self-efficacy could enhance their belief in their capabilities, thereby reducing their anxiety toward math. Similar negative correlation was also observed in the study of Rozgonjuk et al. (20), where the authors suggested that since mathematics self-efficacy plays a significant role in MA, boosting self-efficacy could be an effective strategy for reducing MA. In this context, environmental factors, such as teacher support is crucial. Research has shown that student-perceived math teacher support influences MA through the mediation of the teacher–student relationship and math self-efficacy (21, 22). By fostering positive teacher–student interactions and enhancing self-efficacy, MA can be effectively reduced through increasing mathematical problem-solving ability (23). Conversely, negative classroom experiences, such as adverse teacher behavior or failures in math, are negatively correlated with students’ self-efficacy (3, 24). Similarly, other environmental factors, such as parental stereotypes, culture, social pressure also found influencing effect on MA (2, 3, 25). When examining cognitive factors, researchers often focus on the interaction between MA and working memory (26, 27). According to existing studies, this interaction is reciprocal: higher levels of MA impair working memory (26, 28), leading to more errors and poorer performance in math (29). This poor performance, in turn, further increases MA, creating a vicious cycle (30). Finally, in terms of pedagogical factors, the most frequently documented influence on MA is teaching or instructional strategies. Studies have shown that a rigid curriculum and traditional instructional strategies can significantly contribute to the development of MA (2, 31). Conversely, incorporating technology-enhanced and modernized approaches to mathematics instruction can create an engaging, active learning environment that reduces anxiety and fosters a more positive attitude toward learning math (32).

In educational contexts, it is widely recognized by educational psychologists to practitioners that MA represents a significant barrier to student success across all academic levels. Over the years, numerous studies have investigated the origins of MA and its associated factors, yet few have delved into the intricate causal relationships that play a critical role in predicting MA. Furthermore, most of the existing research has predominantly focused on Western countries, while limited attention given to developing nations such as Bangladesh. To address this gap and support the advancement of domain knowledge, this study aims to identify and analyze the key factors that contribute in shaping MA within the context of Bangladesh, particularly at the secondary education level.

The remaining sections of this study organized as follows:Section 2 briefly discusses the materials and methods. Section 3 presents the findings step-by-step. Section 4 interprets these results in the context of previous research. Section 5 offers recommendations and policy implications. And section 6 highlights the study’s limitations and suggests future research directions.

## 2 Materials and methods

### 2.1 Conceptual model and hypotheses

**Figure 1:**
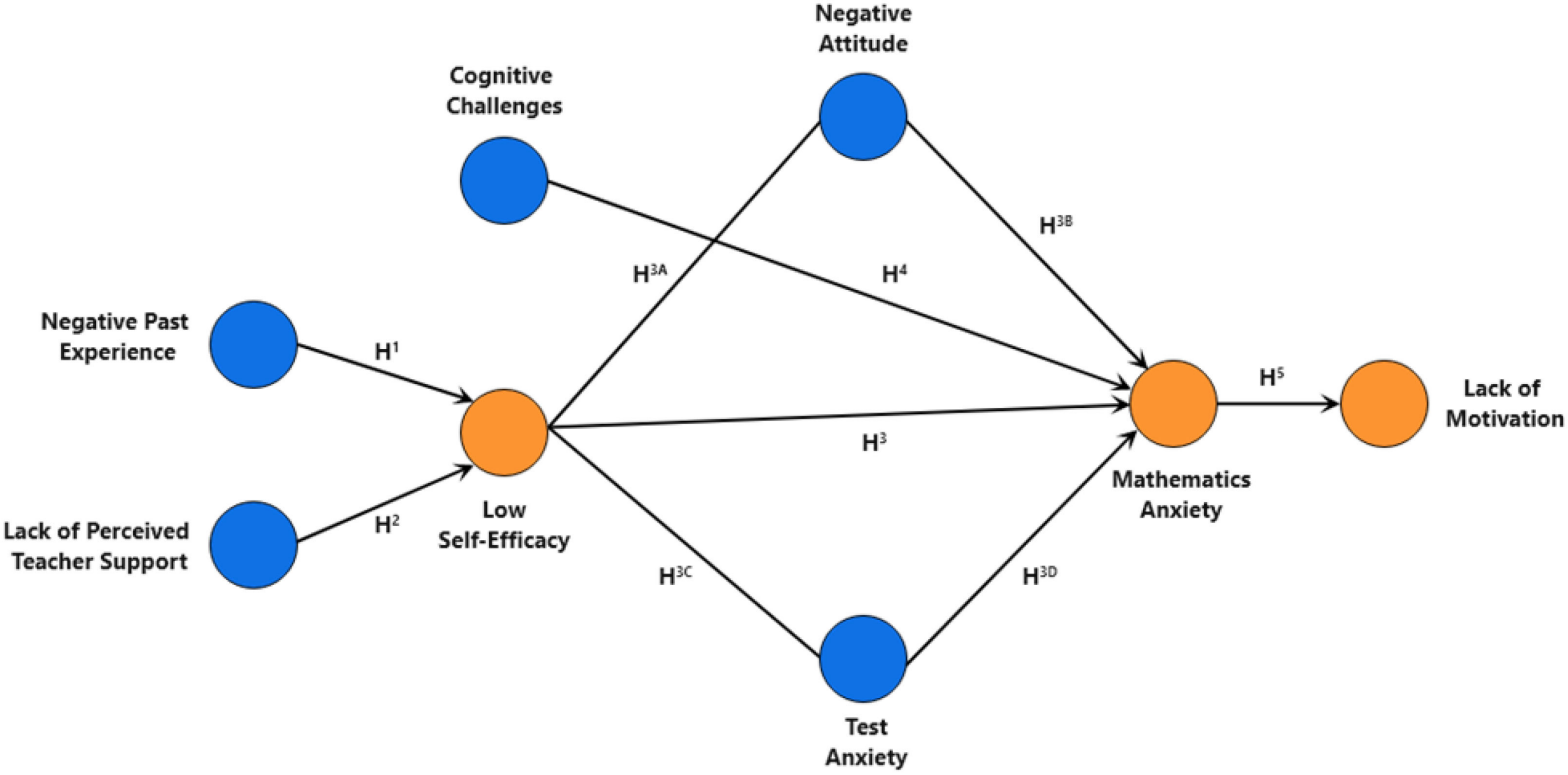
Conceptual model.

This study introduces and empirically evaluates a comprehensive conceptual model designed to identify the factors of mathematics anxiety among secondary school students in Bangladesh (see **Figure 1**). The model elucidates the multi-faceted nature of factors contributing to mathematics anxiety, emphasizing both direct and indirect pathways. The core of the model comprises eight constructs: (i) negative past experiences (NPE) in mathematics learning, (ii) lack of perceived teacher support (LPTS), (iii) cognitive challenges (CC), (iv) low self-efficacy (LSE), (v) negative attitude (NA) towards mathematics, (vi) test anxiety (TA), (vii) mathematics anxiety (MA), and (viii) lack of motivation (LM). Four of these constructs are classified as exogenous (independent latent variables), while the remaining four are endogenous (dependent latent variables). The model posits that negative past experiences and a lack of perceived teacher support directly contribute to the development of low self-efficacy in mathematics among students. This low self-efficacy, further influenced by negative attitudes and test anxiety (mediators), is then hypothesized to directly lead to MA. Additionally, the model proposes that cognitive challenges, specifically working memory difficulties, can directly influence mathematics anxiety. Negative attitudes and test anxiety are also theorized to have a direct effect on increasing MA. Finally, the model suggests that a lack of motivation, manifested as math avoidance, is directly influenced by higher levels of MA. The selection of constructs and the direction of their hypothesized relationships are grounded in theoretical foundations established within the relevant academic literatures (2, 10–13, 17–19, 23, 26, 27, 30–32). Finally, according to the conceptual model illustrated in **Figure 1**, the study hypotheses can be formed as follows:

**H^1^:** Negative Past Experiences in Maths Learning are positively associated with Low Self-Efficacy.

**H^2^:** Lack of Perceived Teacher Support is positively associated with Low Self-Efficacy.

**H^3^:** Low Self-Efficacy is positively associated with Mathematics Anxiety.

**H^3A^:** Low Self-Efficacy is positively associated with Negative Attitudes toward mathematics.

**H^3B^:** Negative Attitudes mediate the relationship between Low Self-Efficacy and Mathematics Anxiety

**H^3C^:** Low Self-Efficacy is positively associated with Test Anxiety.

**H^3D^:** Test Anxiety mediates the relationship between Low Self-Efficacy and Mathematics Anxiety.

**H^4^:** Cognitive Challenges (working memory difficulties) are positively associated with Mathematics Anxiety.

**H^5^:** MA is positively associated with Lack of Motivation (Math Avoidance).

### 2.2 Study design and settings

This study employed a quantitative cross-sectional research design conducted between April 15, 2024, and May 28, 2024. A total of 486 secondary-level students from 89 institutions participated. The cross-sectional design was selected because it analyzes data from a population at a single point in time, rather than following individuals over time (33, 34). This approach is relatively quick and inexpensive, making it an efficient method for generating hypotheses (35). By capturing a snapshot of math anxiety prevalence and examining causal relationships between factors at a specific point in time, this design was ideal and practical for addressing questions about the current state of the phenomenon studied.

### 2.3 Study population and sample size

**Figure 2:**
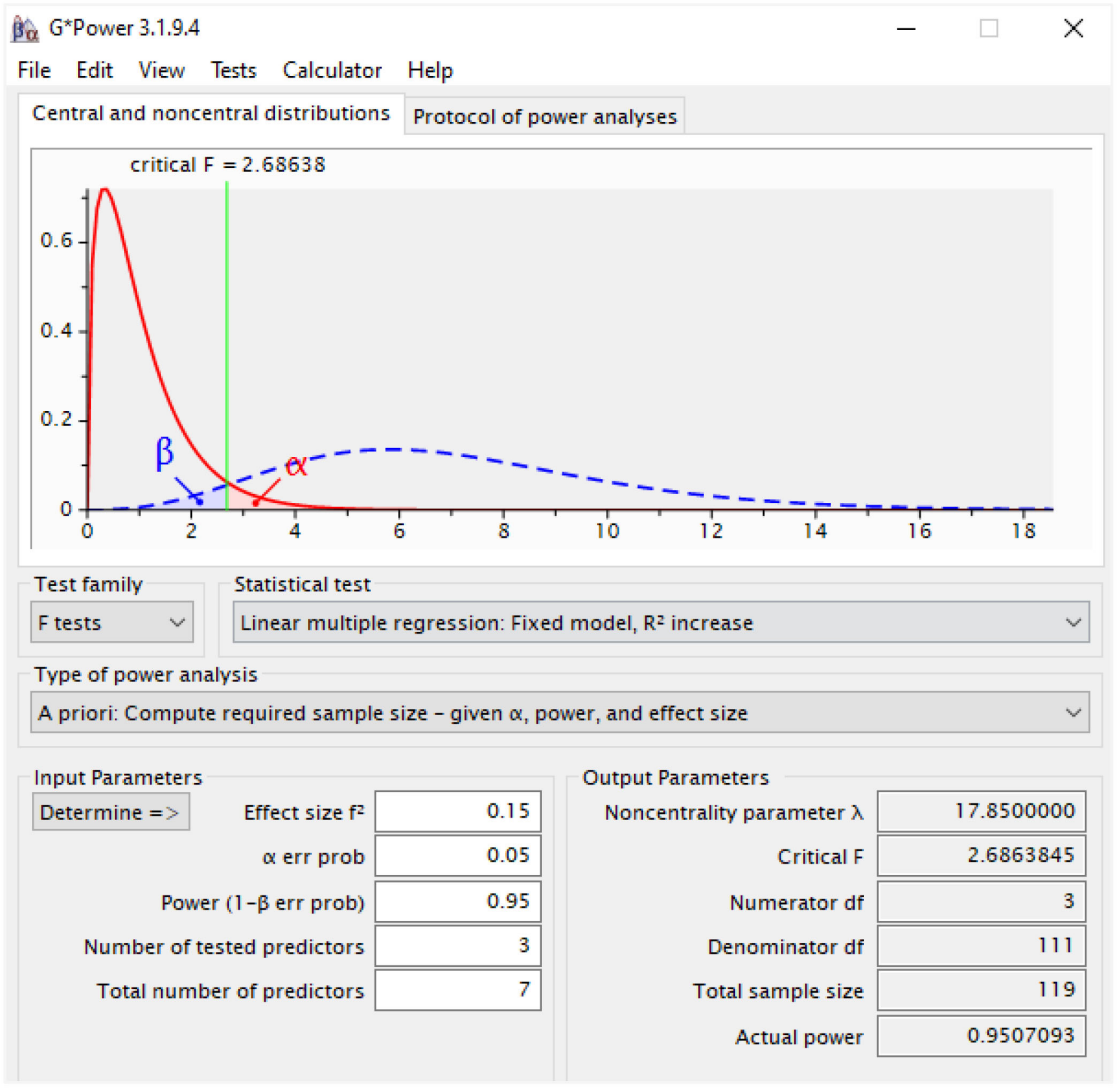
Power result of required sample size.

The target population of this study is secondary-level students in Bangladesh. According to the Bangladesh Bureau of Educational Information and Statistics (BANBEIS), as of 2023, there were 8166188 secondary-level students in 18968 educational institutions in the country (36). The sample size for this research consisted of 486 secondary-level students from 89 institutions, where 297 (61.1%) were male and 189 (38.8%) were female. The participants were between the ages of 12 and 17, with an average age of 14.64 years (standard deviation = 1.93). Although the sample size might seem insufficient compared to the target population, the study utilized Partial Least Squares Structural Equation Modeling (PLS-SEM), a methodology widely known for its statistical robustness even with smaller sample sizes (34, 37). The conventional method for sample size estimation in PLS-SEM is the “10 times rule,” which stipulates that the sample size must be at least 10 times the number of indicators used to measure a construct (38, 39). However, this rule has been criticized for potentially leading to overestimation or underestimation of sample size requirements, as it is not model-specific (34, 40, 41). Therefore, this study employed the G*Power software (version 3.1.9.4) to estimate the required sample size. The G*Power software is widely used and recognized as a reliable tool for sample size determination (34, 37). The input parameters used for the calculation included: Effect size f²=0.15 (medium), α err prob=0.05, Power (1-β err prob) =0.95, Number of tested predictors=3, and Total number of predictors =7. The output showed that the required minimum sample size was 119, where the Actual power was 0.9507. On the other hand, the actual sample size of this study was 486 which was sufficient to ensure standard statistical power (see **Figure 2**).

### 2.4 Data collection instrument and procedure

The data collection of this study occurred between April 15 and May 28, 2024, involving 486 students from 89 secondary-level institutions in Bangladesh. A questionnaire was developed by quantitative research experts to assess the predicting factors of mathematics anxiety. Comprising 55 closed-ended questions, the questionnaire was divided into two primary sections. The first section gathered demographic information, including age, gender, residence, parental education, and mathematics GPA. The second section focused on influencing factors of mathematics anxiety, further subdivided into sub-sections where students rated agreement with statements on a Likert scale of 1 (strongly disagree) to 5 (strongly agree).

Math anxiety (MA) refers specifically to fear and emotional distress related to performing mathematical tasks, whereas test anxiety (TA) is a broader concept that encompasses anxiety experienced during any test-taking situation, regardless of subject. In this study, we measured these constructs separately using distinct scales. MA was assessed using five self-reported items derived from two validated instruments: the modified Abbreviated Math Anxiety Scale (mAMAS) (42) and the Weighted Scoring Based Rating Scale to Identify the Severity Level of Mathematics Anxiety (WSB-MARS) (5). These instruments cover various subdomains, such as computational anxiety and anxiety specifically related to mathematics tests. TA, on the other hand, was measured using items designed based on the to capture general test anxiety experienced during test situations following Jirjees et al. (43).

The validity and reliability of the questionnaire were ensured by content validity and internal consistency reliability respectively. A panel of five domain experts evaluated the questionnaire using a 5-point rating scale for relevance, representativeness, specificity, and clarity, as outlined in study of Ahmmed et al. (37). Cohen’s kappa (k = .87, p<.005***) indicated substantial inter-rater agreement (44). While Cronbach’s alpha coefficient (α=.83, p<.005***) confirmed robust internal consistency reliability (45). Data was collected through both in-person and online surveys, with appropriate approvals and informed consent obtained from participants.

### 2.5 Statistical analysis

To explore the data, two stages of analysis were conducted. Initially, a descriptive analysis using IBM SPSS Statistics 26 (46) was performed. Subsequently, Partial Least Squares Structural Equation Modeling (PLS-SEM) was employed using SmartPLS4 (47) to investigate the key “driver” constructs of mathematics anxiety. PLS-SEM, a sophisticated second-generation multivariate analysis method, is a variance-based technique used for estimating both the structural model (inner model) and the measurement model (outer model) (34, 48). This method is frequently used in exploratory research to develop theory and examine complex relationships between multiple latent variables (48–50). The use of PLS-SEM in this study is justified due to its flexibility in exploring and experimenting with various configurations. Additionally, as noted in prior research, PLS-SEM is often more appropriate than methods such as Covariance-based Structural Equation Modeling (CB-SEM) when the objective is to explore the key “driver” constructs. For statistical accuracy, this study utilized percentile bootstrapping with 5000 sub-samples, employing a fixed seed to ensure the reproducibility of results. A significance level of 0.05 was applied, using a two-tailed test with parallel processing to efficiently generate robust standard errors and significance levels for the path coefficients. This approach ensured the reliability of the parameter estimates and their associated significance.

### 2.6 PLS-SEM parameter settings

To optimize the accuracy and convergence of the model, specific settings in SmartPLS4 were configured. The initial weights for the analysis were set to 1.0, ensuring equal starting values for all indicator variables before optimization. A maximum number of 3000 iterations was allowed to ensure the model had sufficient computational cycles to converge, especially in the case of complex models. The stop criterion was set to 10^7^, ensuring the estimation process would stop once the change in parameter estimates across iterations was below this threshold, indicating precise convergence. All results were standardized, allowing for easy interpretation and comparison of coefficients between constructs within the model. The default Lohmoeller settings were not employed, as custom settings were more appropriate for the data and model characteristics. Additionally, the path weighting scheme was selected, as it is particularly suitable for maximizing explained variance (R²) in endogenous constructs, aligning well with the study’s focus on identifying key drivers of mathematics anxiety.

### 2.7 Ethical consideration

To ensure the research was conducted in accordance with ethical standards, necessary permissions were obtained from the United International University Ethics Review Board (Ref: IREB/2023/018). Informed consent was secured from all participants, and parental or guardian consent was obtained in line with the legal requirements of our jurisdiction. Prior to data collection, parents or legal guardians were invited to the respective schools for a discussion. During this meeting, the research objectives and the questionnaire were shared with them. Written consent was then obtained from those who willingly agreed to participate. This process ensured that both participants and their guardians were fully informed about the study’s purpose, procedures, and the absence of any potential risks. Furthermore, stringent measures were implemented to protect the privacy and confidentiality of participants’ data.

## 3 Results

### 3.1 Demographic information

The study sample consisted of 486 secondary-level students from 89 institutions, comprising 312 (64.2%) males and 174 (35.8%) females. The participants represented a diverse age range, with the majority (89.5%) being between 13 and 15 years old. The mean age was 13.75 years (± 2.72). Regarding socio-economic status, most students (71.6%) came from middle-class families, while 17.7% were from lower-class families, and 10.7% were from upper-class families. Notably, a significant number of students (50.3%) lived with their families, 33.9% lived with friends, and 15.8% resided alone. In terms of math anxiety, 41.4% of the total sample experienced high levels of MA, 47.5% had moderate levels, and 11.1% exhibited low levels of MA. Among male students, 20% had low MA, 51.2% had moderate MA, and 28.7% experienced high MA, with an average MA score of 12.34 (± 4.401). In contrast, female students reported higher anxiety levels: only 6.7% had low MA, while 42.7% had moderate MA, and 50.7% had high MA. The average MA score among females was 15.17 (± 4.864), which was significantly higher than that of their male counterparts. These findings highlight a noticeable gender difference in math anxiety, with female students being more prone to higher anxiety levels.

### 3.2 Measurement model evaluation

The first step in analyzing PLS-SEM results is to evaluate the reflective measurement model, ensuring the reliability and validity of the construct measures to support their inclusion in the path model (50). According to previous studies (34, 48, 49), the standard assessment criteria for this evaluation include three key aspects: internal consistency reliability, convergent validity, and discriminant validity.

#### 3.2.1 Internal consistency reliability

The concept of internal consistency refers to the degree of similarity or homogeneity among the observed indicator variables. Generally, Cronbach’s alpha is used to assess internal consistency reliability (51). This measure estimates reliability based on the inter-correlations of the observed indicators, assuming all indicators have equal reliability and equal outer loadings on the construct. However, in PLS-SEM, indicators are prioritized based on their individual reliability. Consequently, composite reliability is the recommended criterion for measuring internal consistency in PLS-SEM (48). According to the previous studies, Composite reliability values between 0.60 and 0.70 are generally considered acceptable, while those between 0.70 and 0.90 indicate satisfactory internal consistency in exploratory studies (34, 50). As **Table 1** demonstrates, all constructs in our analysis surpassed the recommended threshold of 0.60, confirming the adequate internal consistency of the measurement model.

**Table 1:** Validity and reliability of measurement model.

Constructs	Indicators	Mean Loading	STDEV	AVE	P values	Internal consistency reliability		
						Cronbach’s alpha	Composite Reliability	
							rho_a	rho_c
Cognitive Challenges	CC01	0.816	0.017	0.653	0.000	0.866	0.885	0.903
	CC02	0.848	0.013		0.000			
	CC03	0.859	0.014		0.000			
	CC04	0.840	0.016		0.000			
	CC05	0.658	0.034		0.000			
Lack of Motivation	LM01	0.834	0.024	0.672	0.000	0.841	0.871	0.891
	LM02	0.870	0.016		0.000			
	LM03	0.802	0.029		0.000			
	LM04	0.763	0.031		0.000			
Lack of Perceived Teacher Support	LPTS01	0.788	0.034	0.681	0.000	0.885	0.908	0.914
	LPTS02	0.751	0.032		0.000			
	LPTS03	0.857	0.019		0.000			
	LPTS04	0.843	0.019		0.000			
	LPTS05	0.875	0.019		0.000			
Low Self-Efficacy	LSE01	0.790	0.018	0.622	0.000	0.847	0.852	0.891
	LSE02	0.705	0.030		0.000			
	LSE03	0.842	0.016		0.000			
	LSE04	0.781	0.021		0.000			
	LSE05	0.820	0.018		0.000			
Mathematics Anxiety	MA01	0.843	0.015	0.695	0.000	0.890	0.891	0.919
	MA02	0.811	0.017		0.000			
	MA03	0.846	0.014		0.000			
	MA04	0.810	0.022		0.000			
	MA05	0.855	0.016		0.000			
Negative Attitude	NA01	0.874	0.010	0.640	0.000	0.859	0.884	0.898
	NA02	0.855	0.014		0.000			
	NA03	0.834	0.015		0.000			
	NA04	0.730	0.029		0.000			
	NA05	0.689	0.032		0.000			
Negative Past Experience	NPE01	0.695	0.036	0.527	0.000	0.782	0.790	0.848
	NPE02	0.790	0.028		0.000			
	NPE03	0.731	0.040		0.000			
	NPE04	0.702	0.055		0.000			
	NPE05	0.694	0.053		0.000			
Text Anxiety	TA01	0.850	0.018	0.669	0.000	0.865	0.905	0.907
	TA02	0.480	0.052		0.000			
	TA03	0.886	0.011		0.000			
	TA04	0.887	0.011		0.000			
	TA05	0.904	0.009		0.000			

#### 3.2.2 Convergent validity

Convergent validity refers to the degree to which a measure correlates positively with alternative measures of the same construct. It implies that measures with similar or identical constructs should be substantially related. In PLS-SEM, convergent validity is typically assessed using two criteria: Average Variance Extracted (AVE) and outer loadings. The recommended benchmarks are that outer loadings should be at least 0.708 and AVE values should be at least 0.50 (38). As shown in **Table 1**, all constructs have AVEs exceeding 0.5, indicating that each construct explains over 50% of the variance in its items. Likewise, all outer loadings met the recommended threshold, except for TA02 (outer loading = 0.480), which was subsequently removed to improve the overall AVE. So, it can be concluded that the measurement model has achieved the required level of convergent validity.

#### 3.2.3 Discriminant validity

Discriminant validity reflects the extent to which a construct is distinguishable from other constructs. The main objective of establishing discriminant validity is to ensure that a reflective construct exhibits stronger correlations with its indicators as compared to other constructs. To assess discriminant validity, scholars commonly use the Heterotrait-monotrait ratio (HTMT) and Fornell-Larcker criterion (52). According to exiting studies, HTMT value should be less than 0.90 to establish discriminant validity between reflective constructs (34, 53). As evident in **Table 2**, the HTMT of all constructs met the recommended threshold. On the other hand, Fornell-Larcker criterion involves comparing the square root of the average variance extracted (AVE) values of each construct with its correlations with other constructs within the same model (54). Here, as per the existing studies, the square root of each construct’s AVE should be greater than its highest correlation with any other construct (34). As shown in **Table 3**, the findings support the suggested threshold, thereby confirming the discriminant validity of the reflective construct.

**Table 2:** Heterotrait-monotrait ratio (HTMT).

	CC	LM	LPTS	LSE	MA	NA	NPE	TA
Cognitive Challenges (CC)	–							
Lack of Motivation (LM)	0.438							
Lack of Perceived Teacher Support (LPTS)	0.350	0.369						
Low Self-Efficacy (LSE)	0.596	0.478	0.346					
Mathematics Anxiety (MA)	0.659	0.419	0.262	0.864				
Negative Attitude (NA)	0.544	0.492	0.415	0.777	0.824			
Negative Past Experiences (NPE)	0.566	0.341	0.443	0.376	0.407	0.303		
Test Anxiety (TA)	0.747	0.618	0.282	0.813	0.834	0.698	0.561	–

**Table 3:** Fornell-Larcker criterion.

	CC	LM	LPTS	LSE	MA	NA	NPE	TA
Cognitive Challenges (CC)	0.808							
Lack of Motivation (LM)	0.401	0.819						
Lack of Perceived Teacher Support (LPTS)	0.313	0.347	0.825					
Low Self-Efficacy (LSE)	0.520	0.431	0.323	0.789				
Mathematics Anxiety (MA)	0.590	0.384	0.249	0.751	0.833			
Negative Attitude (NA)	0.475	0.427	0.359	0.670	0.742	0.800		
Negative Past Experiences (NPE)	0.445	0.319	0.369	0.328	0.360	0.262	0.726	
Test Anxiety (TA)	0.667	0.526	0.271	0.706	0.736	0.608	0.479	0.82

### 3.3 Structural model evaluation

Once the validity and reliability of the measurement model have been confirmed, the next step in analyzing PLS-SEM results is to evaluate the structural model. The main goal at this stage is to examine the model’s predictive power and the relationships between constructs (37, 50, 53). To assess the structural model, the standard criteria include three key aspects: collinearity assessment, evaluation of structural model path coefficients, and the coefficient of determination (R²).

#### 3.3.1 Collinearity statistics

Since the path coefficients in the structural model are based on OLS regressions, it is crucial to check for collinearity problems to avoid biased regression outcomes. Collinearity is determined by the construct’s variance inflation factor (VIF) value (34, 38). To prevent collinearity issues, previous studies recommend that VIF value should be above 0.20 and below 5 (50, 53). As shown in **Table 4**, all constructs’ VIF values fall within the recommended range, indicating that the structural model does not have collinearity issues.

**Table 4:** Collinearity assessment.

	VIF	Higher than 0.20 and lower than 5
CC -> MA	1.833	Yes
LPTS -> LSE	1.158	Yes
LSE -> MA	2.447	Yes
LSE -> NA	1.000	Yes
LSE -> TA	1.000	Yes
MA -> LM	1.000	Yes
NA -> MA	1.957	Yes
NPE LSE	1.158	Yes
TA -> MA	2.740	Yes

#### 3.3.2 Path coefficients (direct effects)

The structured model’s path coefficients were evaluated using bootstrapping with 5000 sub-samples, a two-tailed test, and a significance level of 0.05. As shown in **Table 5**, the proposed relationships were found to be statistically significant, providing empirical support for the conceptual model. Each path coefficient indicates the strength and significance of the hypothesized relationships between the constructs. For instance, the direct paths from LPTS and NPA to LSE were significant, with coefficients of β = 0.234 (t = 5.440, p < 0.001) and β = 0.241 (t = 4.914, p < 0.001), respectively. This underscores the crucial role of teacher support and past experiences in shaping students’ self-efficacy, supporting H^1^, and H^2^. Furthermore, LSE demonstrated substantial direct effects on NA, TA, and MA, with coefficients of β = 0.670 (t = 25.249, p < 0.001), β = 0.706 (t = 31.694, p < 0.001), and β = 0.284 (t = 6.704, p < 0.001), respectively. These findings support hypotheses H^3A^, H^3C^, and H^3^, highlighting LSE’s central role in the model. NA and TA also significantly contributed to MA, with coefficients of β = 0.347 (t = 9.063, p < 0.001) and β = 0.251 (t = 5.913, p < 0.001), respectively, thus supporting hypotheses H^3C^. Notably, the path from CC to MA was also statistically significant with a coefficient of β = 0.110 (t = 2.659, p = 0.008), suggesting that difficulties in working memory directly contribute to increased MA, supporting H^4^. Finally, MA also significantly influenced LM, as indicated by a coefficient of β = 0.384 (t = 9.939, p < 0.001), supporting H^5^. The high t-statistics and low p-values across all paths indicate the model’s validity in explaining the multifaceted contributors to MA among the secondary level students in Bangladesh.

**Table 5:** Path coefficients (direct effects).

	Original sample (O)	Sample mean (M)	Standard deviation (STDEV)	T statistics (|O/STDEV|)	P values	Confidence Intervals (CIs)	
						2.5%	97.5%
CC -> MA	0.110	0.111	0.041	2.659	0.007	0.030	0.193
LPTS -> LSE	0.234	0.235	0.043	5.440	0.000	0.152	0.321
LSE -> MA	0.284	0.287	0.042	6.704	0.000	0.204	0.370
LSE -> NA	0.670	0.671	0.027	25.249	0.000	0.616	0.720
LSE -> TA	0.706	0.707	0.022	31.694	0.000	0.656	0.745
MA -> LM	0.384	0.388	0.039	9.939	0.000	0.310	0.463
NA -> MA	0.347	0.345	0.038	9.063	0.000	0.275	0.424
NPA -> LSE	0.241	0.247	0.049	4.914	0.000	0.152	0.340
TA -> MA	0.251	0.250	0.043	5.913	0.000	0.161	0.324

#### 3.3.3 Indirect effects

The structured model also evaluated the specific and total indirect effects (see **Table 6**), highlighting the mediating roles of NA and TA between LSE and MA. A mediating effect is developed when a third variable or construct (in our case, TA and NA) intervenes between two other related constructs (in our case, LSE and MA). To understand the size of the mediating effect, variance accounted for (VAF) is typically suggested by the scholars (48) that determines the size of the indirect effect in relation to the total effect using the following formula (1):

**Table 6:** Indirect effects.

Specific indirect effects								Confidence Intervals (CIs)	
	Original sample (O)	Sample mean (M)	Standard deviation (STDEV)	T statistics (|O/STDEV|)	P values	VAF Value	Mediation Type	2.5%	97.5%
LSE -> NA -> MA	0.237	0.236	0.024	9.787	0.000	45.4%	Partial mediation	0.186	0.284
LSE -> TA -> MA	0.171	0.170	0.029	5.795	0.000	37.4%	Partial mediation	0.114	0.229
Total indirect effect									
LSE -> MA	0.410	0.408	0.040	10.232	0.000			0.327	0.486


(1) $$\text{VAF= }\frac{\text{Indirect Effect}}{\text{Total Effect}}=\frac{{\text{β}}_{\text{indirect}}}{{\text{β}}_{\text{direct}}{\text{ + β}}_{\text{indirect}}}$$

As per the guideline of Hair et al. (48), if the VAF value is less than 20%, and it can be concluded that (almost) no mediation takes place. On the other hand, if the VAF value is larger than 20% and less than 80%, it can be characterized as partial mediation, meaning, a part of the effect of the independent variable (IV) on the dependent variable (DV) is transmitted through the mediator, but the IV still has a direct effect on the DV even after accounting for the mediator. In contrast, when VAF has very large outcomes of above 80%, it can be assuming a full mediation, meaning, indirect effect of an IV on a DV through a mediator is so strong that the direct effect of the IV on the DV becomes non-significant. In this case, the specific indirect effect of LSE on MA through NA was significant, with a coefficient of β = 0.237 (t = 9.787, p < 0.001) and the VAF was 45.4%, indicating that nearly half of the effect of LSE on MA was transmitted through NA, demonstrating a partial mediation effect (48). Similarly, the indirect effect of LSE on MA through TA was also significant, with a coefficient of β = 0.171 (t = 5.795, p < 0.001). The VAF was 37.4%, suggesting that a significant portion of the effect of LSE on MA is mediated by TA, also indicating partial mediation (48). Furthermore, the total indirect effect of LSE on MA, summing the indirect effects through both NA and TA, was substantial, with a coefficient of β = 0.410 (t = 10.232, p < 0.001). The high t statistic and very low p-value confirm the significance of the total indirect effect, emphasizing the substantial impact of LSE on MA through these mediators. So, the findings support the hypotheses H^3B^ and H^3D^.

#### 3.3.4 Total effects (sum of direct & indirect effects)

**Figure 3:**
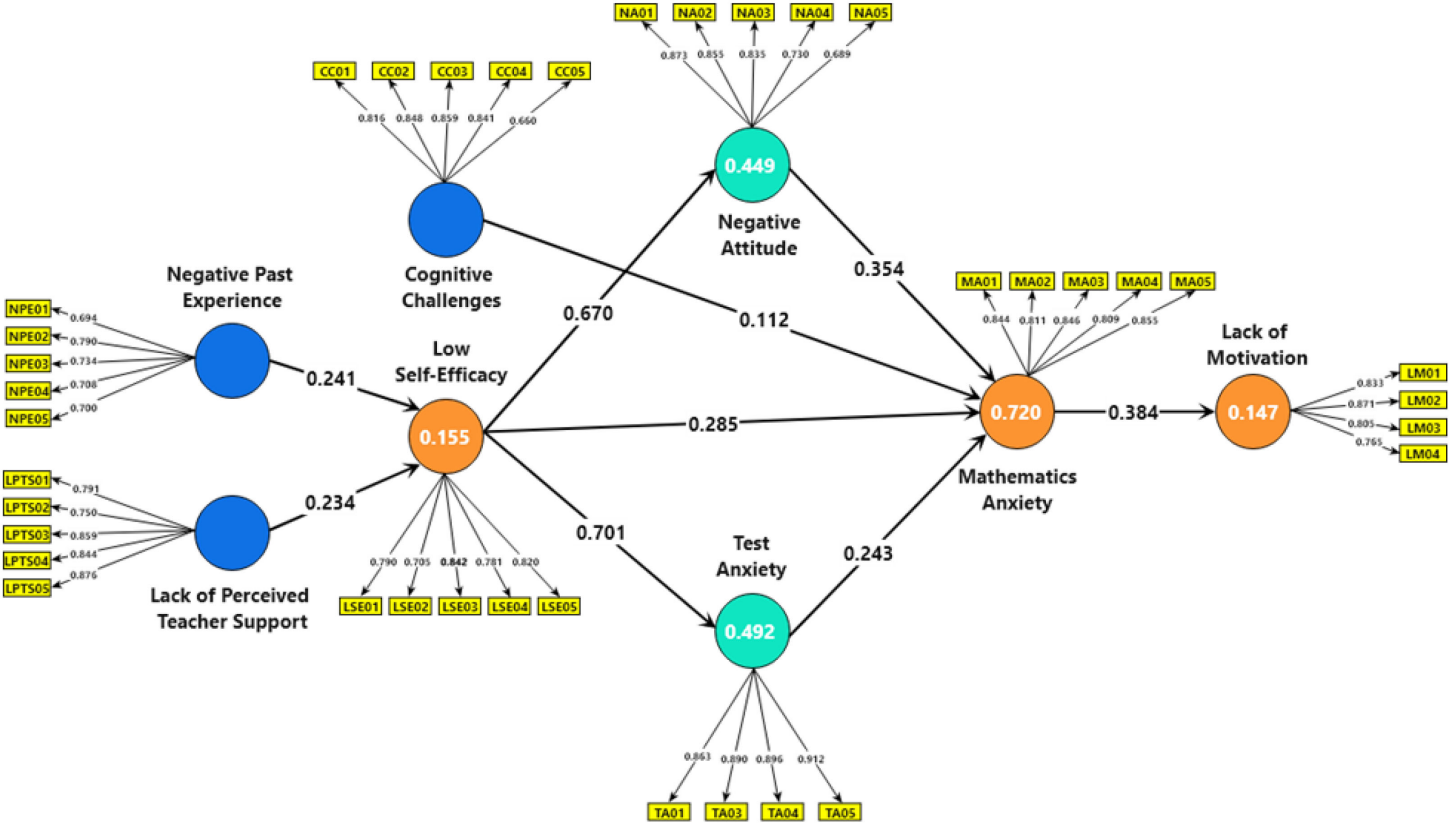
Final model.

**Figure 4:**
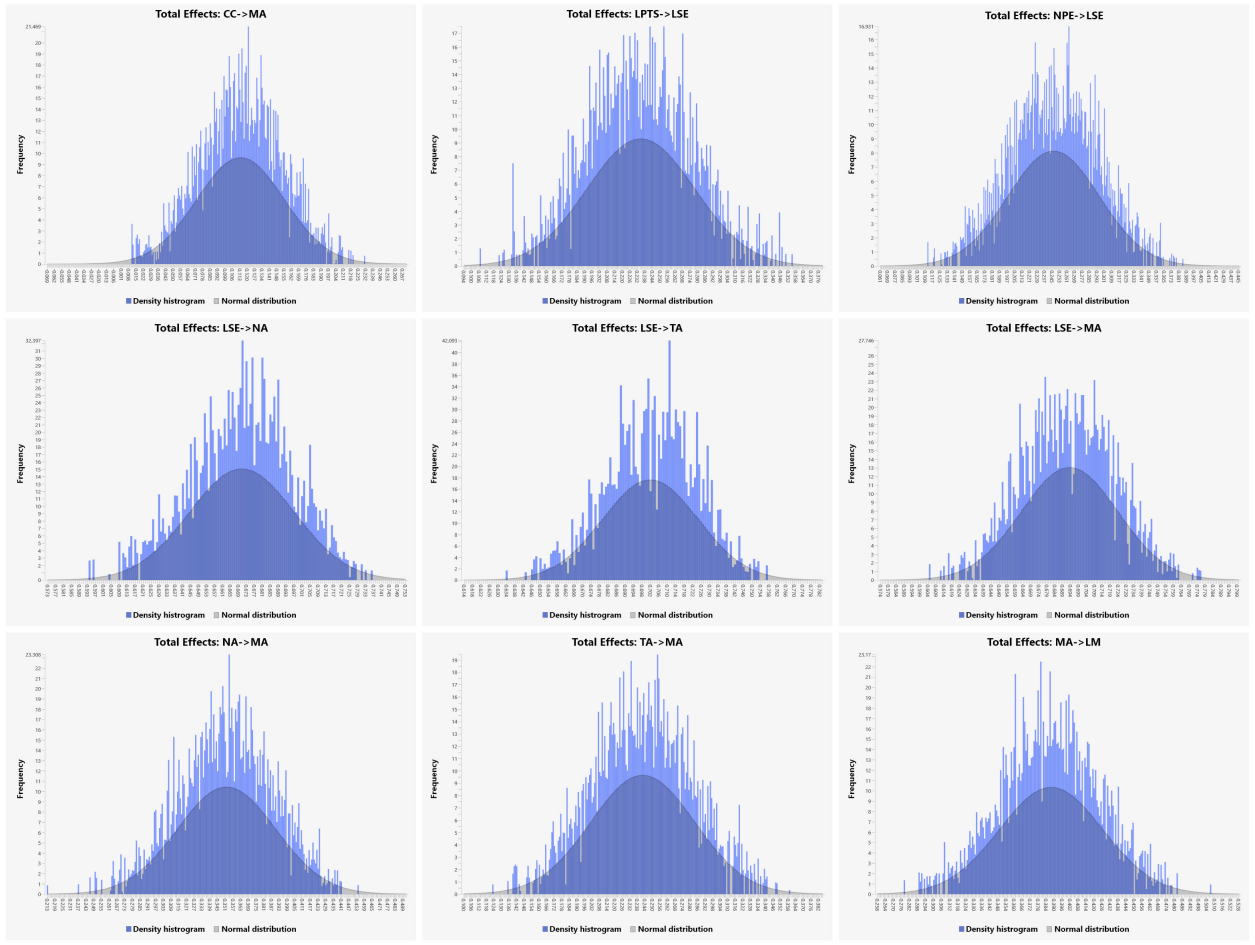
Histograms of the total effect of the factors.

As shown in **Table 7**, **Figure 3**, and **Figure 4**, the total effects analysis revealed that all examined relationships were statistically significant, underscoring the key contributors to mathematics anxiety among secondary-level students in Bangladesh. NPE had a significant total effect on LSE, with a coefficient of β = 0.241 (t = 4.914, p < 0.001), indicating that negative past experiences significantly lowered students’ self-efficacy. Similarly, LPTS significantly influenced LSE, with a coefficient of β = 0.234 (t = 5.440, p < 0.001), suggesting that insufficient teacher support also contributed to reduced self-efficacy. The total effect of LSE on MA was substantial, with a coefficient of β = 0.694 (t = 22.695, p < 0.001), highlighting the pivotal role of self-efficacy in the development of MA. CC, specifically working memory difficulties, were also found to significantly impact MA, with a coefficient of β = 0.110 (t = 2.659, p = 0.008), indicating that cognitive difficulties contribute to increased anxiety. LSE was further found to significantly influence both NA and TA, with coefficients of β = 0.670 (t = 25.249, p < 0.001) and β = 0.706 (t = 31.694, p < 0.001), respectively. This suggests that LSE leads to more NA towards mathematics and higher levels of test anxiety. Both NA and TA then significantly contributed to MA, with coefficients of β = 0.354 (t = 9.063, p < 0.001) and β = 0.243 (t = 5.913, p < 0.001), respectively, underscoring their roles in exacerbating MA. Finally, MA significantly affected LM, with a coefficient of β = 0.384 (t = 9.939, p < 0.001), indicating that higher levels of MA correlate with LM to engage in mathematics-related activities.

**Table 7:** Total effects.

	Original sample (O)	Sample mean (M)	Standard deviation (STDEV)	T statistics (|O/STDEV|)	P values	Confidence Intervals (CIs)	
						2.5%	97.5%
CC -> MA	0.112	0.111	0.041	2.659	0.008	0.030	0.193
LPTS -> LSE	0.234	0.235	0.043	5.440	0.000	0.152	0.321
LSE -> MA	0.694	0.695	0.031	22.695	0.000	0.634	0.754
LSE -> NA	0.670	0.671	0.027	25.249	0.000	0.616	0.720
LSE -> TA	0.701	0.707	0.022	31.694	0.000	0.656	0.745
MA -> LM	0.384	0.388	0.039	9.939	0.000	0.310	0.463
NA -> MA	0.354	0.345	0.038	9.063	0.000	0.275	0.424
NPE-> LSE	0.241	0.247	0.049	4.914	0.000	0.152	0.340
TA -> MA	0.243	0.250	0.043	5.913	0.000	0.161	0.324

#### 3.3.5 Coefficient of determination (R^2^)

The coefficient of determination (R²) for the endogenous constructs is used to evaluate the model’s explanatory power. R² values range from 0 to 1, with higher values indicating greater explanatory strength. As per Hair et al. (49), R² values of 0.75, 0.50, and 0.25 are considered substantial, moderate, and weak, respectively (34, 48). **Table 8** presents the R² values for LM, LSE, MA, NA, and TA as 0.147, 0.155, 0.720, 0.449, and 0.492, respectively, demonstrating the model’s satisfactory explanatory power.

**Table 8:** Coefficient of determination (R^2^).

	R-square	R-square adjusted	explanatory power
LM	0.147	0.146	weak
LSE	0.155	0.151	weak
MA	0.720	0.718	moderate
NA	0.449	0.448	moderate
TA	0.492	0.491	moderate

#### 3.3.6 PLSpredict (PLS path model estimations)

The **Table 9** presents the PLSpredict path model estimations, utilizing the algorithm developed by Shmueli et al. (55), which evaluates predictive performance through Q²predict, RMSE (Root Mean Square Error), and MAE (Mean Absolute Error) metrics. The constructs assessed include LM, LSE, MA, NA, and TA. The Q²predict values, reflecting predictive accuracy, range from 0.100 for LM to 0.208 for MA, with the MA constructs demonstrating the highest predictive performance. The RMSE values, which measure the variability of prediction errors, indicate that the MA model exhibits superior accuracy with the lowest RMSE of 0.893. The MAE values, representing the average magnitude of prediction errors, identify the LSE model as having the smallest error at 0.737. Overall, the MA model is distinguished by its highest Q²predict and lowest RMSE, while the LSE model is notable for its minimal MAE, suggesting that these constructs provide superior predictive performance according to the PLSpredict algorithm.

**Table 9:** PLSpredict LV summary.

	Q²predict	RMSE	MAE
LM	0.100	0.952	0.818
LSE	0.141	0.930	0.737
MA	0.208	0.893	0.739
NA	0.122	0.941	0.767
TA	0.170	0.914	0.778

## 4 Discussion

This study aimed to identify and analyze the key factors contributing to MA among secondary-level students in Bangladesh, using a Partial Least Squares Structural Equation Modeling (PLS-SEM) approach. The findings offer valuable insights into the complex interplay of factors that exacerbate MA, reinforcing the results of previous research while contributing new evidence from the context of a developing country. The results highlight a moderate but significant impact of cognitive challenges (CC), particularly working memory difficulties, on MA (β = 0.110, t = 2.659, p = 0.008). Although extensive research has established a negative relationship between MA and working memory (56, 57), no prior study has empirically identified working memory difficulties as a predictor of MA. Therefore, our findings can be interpreted as follows: working memory difficulties contribute to poor mathematical performance, which, in turn, may trigger MA, consistent with the Deficit Theory (58). In this sense, our findings align with existing literature, which suggests that cognitive overload impairs mathematical performance, thereby exacerbating anxiety (26, 28). We also found that both Negative Past Experiences (NPE) and Lack of Perceived Teacher Support (LPTS) had significant positive causal effects on Low Self-Efficacy (LSE), with coefficients of β = 0.241 (t = 4.914, p < 0.001) and β = 0.234 (t = 5.440, p < 0.001), respectively. These results indicate that negative past experiences and insufficient teacher support significantly contribute to reduced student self-efficacy. These findings are consistent with those of Shukla et al. (24) and Dowker et al. (3), who reported that negative classroom experiences, such as adverse teacher behavior or failures in mathematics, are negatively correlated with students’ self-efficacy. Furthermore, the strong positive association between low self-efficacy (LSE) and MA (β = 0.694, t = 22.695, p < 0.001), demonstrating that students who doubt their mathematical abilities are more likely to experience MA. Notably, the total indirect effect of LSE on MA, mediated through both Negative Attitude (NA) towards math and Test Anxiety (TA), was substantial (β = 0.410, t = 10.232, p < 0.001). This finding underscores the significant impact of LSE on MA through these mediators (NA and TA), aligning with studies by Khasawneh et al. (10) and Rozgonjuk et al. (20), which highlight the critical role of self-efficacy in managing mathematical anxiety. Additionally, the study identified that both NA toward mathematics and TA independently have a significant impact on MA (math achievement). Finally, this study also revealed that MA has a causal positive relationship with lack of motivation (LM) in math learning. The connection between math anxiety and decreased motivation is also evident in previous studies (59–62). This is because the anxiety associated with math can lead to avoidance behaviors, reduced confidence in their math abilities, and adversely affects their future math performance (60).

## 5 Recommendations andpolicy implications

Based on the findings of this study, several key recommendations and policy implications are suggested to address and mitigate MA among secondary-level students in Bangladesh:

A. Enhance Teacher Training: Prioritize the development and implementation of teacher training programs that focus on creating supportive classroom environments and employing teaching methods that build student confidence and self-efficacy in mathematics.

B. Invest in Professional Development: Ensure ongoing professional development for teachers, emphasizing the latest research in educational psychology, teaching strategies, and tools for effectively managing classroom dynamics and student anxiety.

C. Real-World Application: Integrate activities and curricula that highlight the practical applications of mathematics in everyday life. Foster a growth mindset by encouraging students to view mathematical challenges as opportunities for growth.

D. Support for Students with Cognitive Challenges: Provide additional support for students facing cognitive challenges, particularly related to working memory. This could involve personalized tutoring, memory aids, and teaching strategies that break down complex problems. Leveraging educational technologies can support cognitive development and offer interactive learning experiences.

E. Comprehensive Support Systems: Develop integrated support systems including counselors, psychologists, and special educators to address the diverse needs of students.

F. Boost Student Motivation: Employ strategies to increase student motivation and engagement in mathematics, such as gamification, setting achievable goals, and providing timely, constructive feedback.

## 6 Study limitations and future research directions

Despite potential findings, this study acknowledges several limitations. Firstly, a significant limitation is the exclusion of several potential factors, such as gender, parental attitudes, and instructional strategies, due to the research’s limited scope. Including these variables would have complicated the exploration, as examining all such factors in a single study would be challenging, and incorporating mediator factors would add further complexity to the structural model. Secondly, the use of self-report measurements could introduce potential bias, as this method is often criticized for the difficulty respondents may face in making conscious judgments and the tendency to provide socially desirable answers (63). However, we attempted to mitigate this limitation by ensuring students through informed consent that their responses would remain confidential. Thirdly, in this study, we focused primarily on self-reported cognitive difficulties (working memory difficulties) rather than objective cognitive testing. We recognize that the lack of objective cognitive assessments is a limitation, as it would provide a more comprehensive understanding of the participants’ actual cognitive performance. So, in future, objective cognitive assessment is suggested to gain a more nuanced understanding of the cognitive challenges faced by individuals with MA. Fourthly, there is a scarcity of previous studies in developing countries like Bangladesh that explore the complex causal relationships between factors related to MA. This has made it challenging to adequately compare this study’s findings with other studies in the context of Bangladesh. Fifthly, the study’s quantitative cross-sectional design, with data collected over a short period, limits the ability to fully understand and generalize the relationships between the factors. Finally, this study single-level PLS-SEM due to the nature of the data, which reflects individual-level factors influencing MA. However, a multilevel design, especially considering hierarchical relationships (e.g., student-teacher or school-level effects) could be utilized for more precise finings. So, future research should include longitudinal studies, both quantitative and qualitative with multi-level SEM design, to enhance the understanding and generalizability of these relationships.

## 7 Conclusion

This study unveiled a complex interplay of factors contributing to mathematics anxiety (MA) among Bangladeshi secondary students. By employing a robust quantitative approach, this study demonstrates that negative past experiences, perceived lack of teacher support, and cognitive challenges, such as working memory difficulties, significantly contribute to MA. These factors, combined with low self-efficacy, negative attitudes, and test anxiety further create a complex web that exacerbates MA and undermines students’ motivation and performance in mathematics. Addressing these issues requires a multifaceted approach, including enhanced teacher training, professional development, and the integration of practical applications of mathematics in curricula. Additionally, supporting students with cognitive challenges and fostering comprehensive support systems are essential for mitigating MA. By implementing these recommendations, educational authorities can better support students, improve their mathematical experiences, and ultimately enhance their future performance in mathematics.

## Data Availability

The raw data supporting the conclusions of this article will be made available by the authors, without undue reservation.
